# Diagnostic potential of ultrasound in carpal tunnel syndrome with different etiologies: correlation of sonographic median nerve measures with electrodiagnostic severity

**DOI:** 10.1186/s12891-019-3010-5

**Published:** 2019-12-29

**Authors:** Basant Elnady, Elsayed M. Rageh, Tohamy Ekhouly, Sabry M. Fathy, Mohamed Alshaar, El Saeed Fouda, Mohammed Attar, Ahmed M. Abdelaal, Ahmed El Tantawi, Mohammed M. Algethami, David Bong

**Affiliations:** 1 Department of Rheumatology, Rehabilitation and Physical Medicine, Benha University, Benha, Egypt; 2 Department of Internal Medicine, Al Hada Forces Hospital, Alhada, Saudi Arabia; 3 Department of Rheumatology, Rehabilitation and Physical Medicine, Tanta University, Tanta, Egypt; 4 Department of Radiology, Benha University, Benha, Egypt; 5 Department of Radiology, Al Hada Forces Hospital, Al Hada, Saudi Arabia; 6 Department of Neurology, Al Azhar University, Al Azhar, Egypt; 7 Department of Neurology, Al Hada Forces Hospital, Alhada, Saudi Arabia; 8 Department of Pediatrics, Menoufia University, Shebeen El-Kom, Egypt; 9 Department of Neurosurgery, Salford Royal NHS Foundation Trust, Salford, England; 10 Department of Neurosurgery, Ain Shams University, Ain Shams, Egypt; 11 Department of Neurosurgery, Al Hada Forces Hospital, Al Hada, Saudi Arabia; 12 Instituto Poal de Reumatologia, University of Barcelona, Barcelona, Spain

**Keywords:** Carpal tunnel syndrome, Median nerve, Ultrasound

## Abstract

**Background:**

Carpal tunnel syndrome (CTS) is the commonest entrapment neuropathy. The aim of this study was to assess the accuracy and validity of high resolution musculoskeletal ultrasound (US) in the diagnosis of CTS in the Saudi population.

**Methods:**

Sixty patients were diagnosed clinically to have CTS involving 89 wrists that were confirmed by neurophysiologic studies. Each affected wrist was characterized as idiopathic or associated with either diabetes mellitus or hypothyroidism and were assigned a severity grade based on results of neurophysiologic studies. Seventy-six healthy wrists from fifty age, sex and BMI matched healthy subjects were included in the control group. High resolution ultrasound (US) was performed to assess median nerve cross sectional area distal (CSAd) at the entry to the carpal tunnel and proximally (CSAp) at the level of pronator quadratus muscle with a further calculation of their difference (ΔCSA) and their mean average or CSAd+CSAp/2 (CSApd).

**Results:**

There was a significant difference between both groups regarding mean ± SD of CSAd, CSAp, ∆CSA, and CSApd (*p* = 0.0001). A positive significant correlation was also found between the CSAd, ∆ CSA and the CSApd measurements with neurophysiologic severity grade of CTS (*P* = 0.001). A ∆CSA threshold of 2.5 mm^2^ showed the highest sensitivity and specificity to diagnose CTS in Saudis.

**Conclusion:**

High resolution ultrasound is a valid and accurate diagnostic modality in carpal tunnel syndrome and correlated to CTS severity. A ∆CSA greater than 2.5 mm^2^ is considered a valid diagnostic value for CTS in our Saudi population. CTS in our patients with diabetes tend to have greater median nerve US measurement values.

## Background

Carpal tunnel syndrome (CTS) is the most frequent nerve entrapment neuropathy; it occurs secondary to compression of the median nerve under the flexor retinaculum of wrist joint and leads to an enlargement of its cross-sectional area (CSA) just proximal to the site of entrapment (1).

CTS is considered an idiopathic condition, however, characteristic anatomical variations may participate in development of CTS, such as persistent median artery, or a bifid median nerve (2). It may also result from traumatic injury, inflammatory arthritis such as rheumatoid arthritis, or in association of hypothyroidism, diabetes mellitus, or pregnancy (3, 4).

Early diagnosis is essential to alleviate permanent nerve damage and functional disability. The diagnosis of CTS is usually based on clinical and neurophysiological studies (1).

US is a diagnostic imaging modality is being used more often in daily clinical practice not only to confirm the diagnosis of CTS but also it can detect anatomical variations, nerve shape and space-occupying lesions as tenosynovitis and ganglion cysts (5, 6).

Modern US equipment allows freehand tracing of the circumference of the median nerve proximal to the site of entrapment and calculation of the cross-sectional area (CSA). Unfortunately, there is no clear cut and generally accepted cutoff value of a single CSA measurement to diagnose CTS as different studies have producing widely variable results with various cutoff threshold for establishing the diagnosis of CTS which appear to be affected by group ethnicity. (7–16). Although studies had investigated median nerve CSA at the wrist in CTS patients exclusively with diabetes and hypothyroidism (17–19), a comparison of different US parameters, especially the CSA, in patients with idiopathic CTS versus those with CTS associated with diabetes and hypothyroidism has not been compared in the same study.

Klauser et al., assessed the CSA of median nerve distally at the region of maximal enlargement, usually just proximal to the carpal tunnel, and proximally at the level of pronator quadratus muscle, thereby using the same nerve as its own control, to improve the precision of CTS diagnosis (20).

The aim of this study was to assess the accuracy of ultrasound in diagnosis of CTS in the Saudi population using the method developed by Klauser et al. (i.e. ΔCSA) and also the mean of the proximal and distal (mean CSApd) measurements. The median nerve US measurements were further analyzed to detect any difference in the parameters between idiopathic CTS and CTS associated with diabetes mellitus or hypothyroidism.

## Methods

### Study design

#### Cross sectional cohort, randomized study

##### Study population

Sixty consecutive electrophysiologically confirmed CTS patients presenting to a tertiary Saudi medical center (Al Hada Armed Forces Hospital) rheumatology, neurology, neurosurgery out-patients’ clinics, during a six month period underwent US evaluation and fifty age, sex and body mass index (BMI) matched healthy subjects free from CTS manifestation with negative electrophysiologic studies for CTS from the healthy hospital workers were included as a control group for identical US study.

##### Inclusion criteria

Age > 18 years; right handed patients with paresthesia, numbness or tingling affecting the first three digits and the radial half of the fourth digit, which was confirmed by electrophysiologic study.

##### Exclusion criteria

BMI ≥ 30, past history of traumatic or surgical intervention; arthritis; median nerve injection, history of autoimmune rheumatic disease; bifid median nerve, or intra-articular lesions such as ganglion. Patients with nerve conduction study (NCS) findings suggestive of diabetic neuropathy rather than entrapment neuropathy at the carpal tunnel also were excluded.

#### Electrophysiologic methods

The electrodiagnostic studies were performed according to standard techniques for motor and sensory nerve conduction studies of median and ulnar nerves. Motor study included the determination of conduction velocity, amplitudes and latencies after stimulation of the median nerve. Sensory nerve conduction studies included the antidromic determination of conduction velocity, latencies and amplitude of the sensory nerve action potential of the median nerve. These studies were performed for all participants within 2 weeks before US examination by using a Dantec Key Point electrophysiologic device at a fixed room temperature of 25 °C during the examination of all patients and controls.

Diagnosis of CTS was based on the measurement of the median nerve compound muscle action potential (CMAP) amplitude and distal latency from the abductor pollicis brevis following stimulation 8 cm proximal to the recording electrode and the sensory nerve action potential (SNAP) obtained from the middle finger with ring-type electrodes. The palmar and wrist stimulations were 7 and 14 cm, respectively, proximal to the recording electrode.

Electrodiagnostic studies were done on both hands by expert neurologist blinded to the clinical data and study purpose. Only patients with three of the following criteria were included: median SNAP peak latency > 3.7 ms; a SNAP peak latency of the proximal 7-cm segment greater than the peak latency of the distal 7-cm segment; SNAP amplitude was < 20 μV including a conduction block (a SNAP amplitude drop of > 50% with respect to the proximal stimulation, as compared with that of the distal stimulation); median CMAP distal latency was > 4.2 ms; and CMAP amplitude was < 4.5 mV. CTS was diagnosed in patients with diabetic neuropathy if they met the following criteria: the ratio of the distal motor latency of the median to the ulnar nerve was > 1.5; the ratio of the distal sensory latency of the median to the ulnar nerve was > 1.2; the amplitude ratio of the median SNAP to the ulnar SNAP was < 0.6 (21–25).

The severity of CTS was classified as mild, moderate or severe according to the modified scoring system of Padua et al. (26); Severe (absence of sensory response, abnormal distal motor latency (DML)), Moderate (abnormal sensory nerve conduction velocity (SNCV), abnormal DML), Mild (abnormal SNCV, normal DML). Furthermore, electrophysiologically confirmed CTS patients were stratified as to the etiology: idiopathic, diabetic or hypothyroid.

#### US technique

**Fig. 1:**
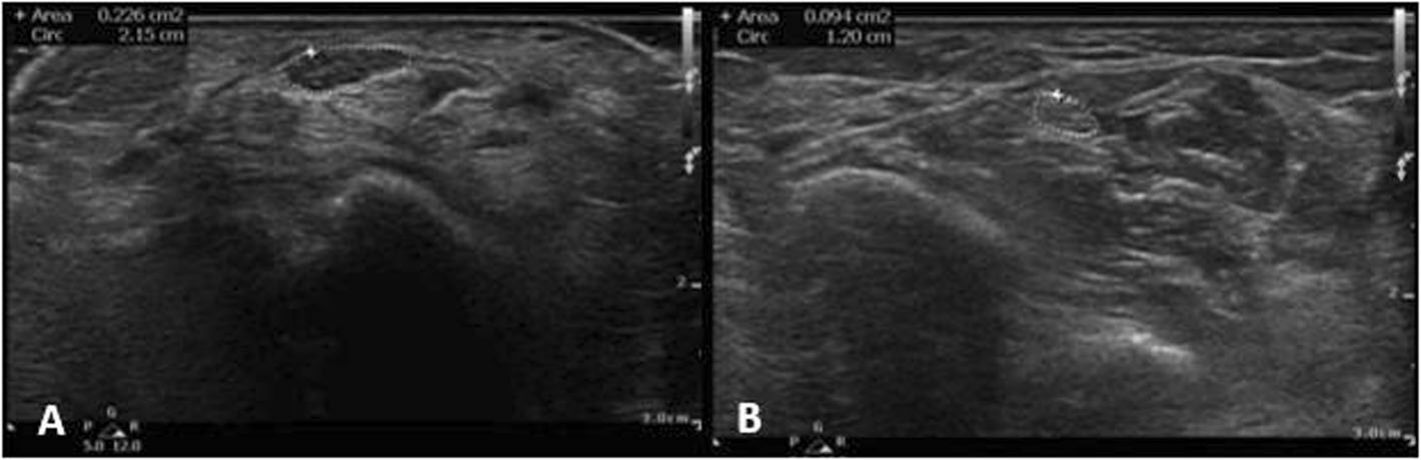
Transverse US image of right median nerve (outlined) at level of carpal tunnel (A) with CSAd is 22.6 mm^2^ and at the level of pronator quadratus muscle(B) with CSAp is 9.4 mm^2^ in a patient with diabetes and CTS

**Fig. 2:**
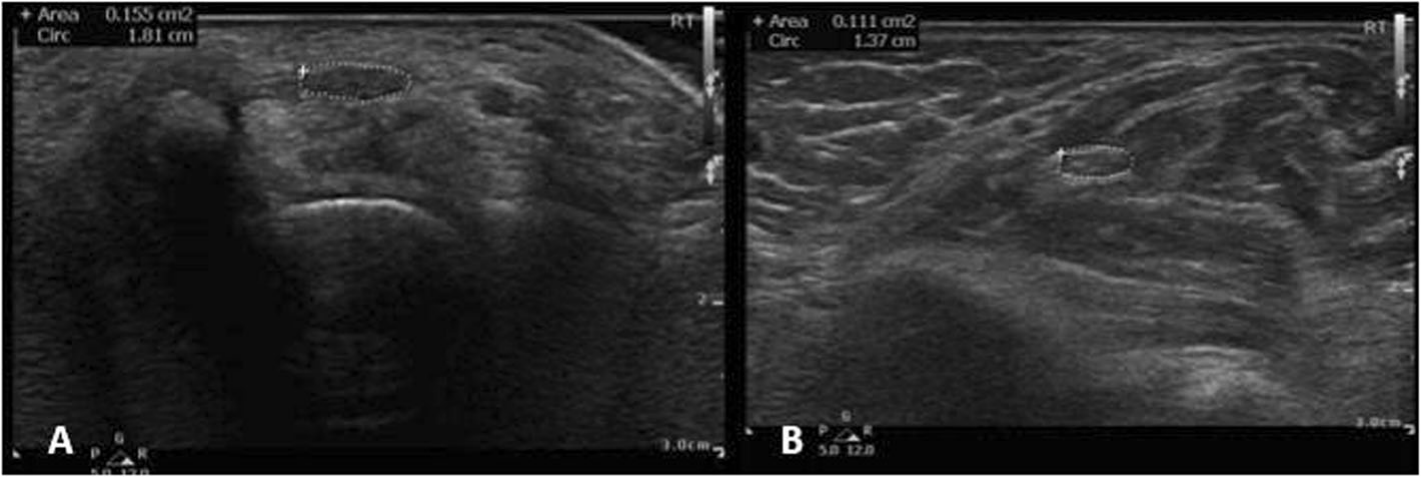
Transverse US image of right median nerve (outlined) at level of carpal tunnel (A) with CSAd is 15.5 mm^2^ and level of pronator quadratus muscle(B) with CSAp is 11.1 mm^2^ in a patient with hypothyroidism and CTS

**Fig. 3:**
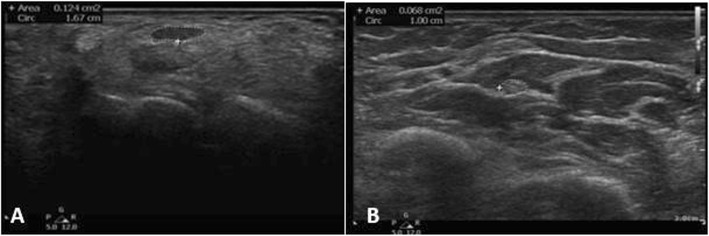
Transverse US image of right median nerve (outlined) at level of carpal tunnel (A) with CSAd is 12.4 mm^2^ and level of pronator quadratus muscle(B) with CSAp is 6.8 mm^2^ in a patient with idiopathic CTS

Sixty consecutive patients with electrophysiologically confirmed CTS and 50 healthy controls with normal electrophysiologic studies of the median nerve were examined by US by two investigators expert radiologist and rheumatologist blinded to the clinical and electrophysiologic results. 89 out of 120 wrists of the patients fulfilled inclusion criteria and were evaluated by US, whereas 76 of 100 wrists of the healthy controls underwent US evaluation (Philips CX50 scanner, using two multi-frequency linear transducers, 6–18 MHz and 5–12 MHz). Transverse US scanning of the median nerve from the distal forearm to the carpal tunnel outlet was performed with measurement of the median nerve CSA distal (CSAd) at its apparent maximal dimension of the thickest part of median nerve at the tunnel (Figs. 1, 2, 3a). Proximal transverse US scanning for CSA proximal (CSAp) measurement was done over the distal third of the pronator quadratus muscle (Figs. 1, 2, 3b). The median nerve was identified between the flexor digitorum superficialis and flexor pollicis longus muscle/myotendinous junction. Then the difference between CSAd and CSAp (ΔCSA) was calculated for each wrist in both patients and controls (20). A mean of the two CSA’s on each US study was calculated by adding the tabulated values of CSAd and CSAp and dividing the sum by two to get the mean CSAdp (CSAd+CSAp/2).

We used the direct measurement technique, after capturing the appropriate image. Free-hand tracing was done with a continuous line around the median nerve fascicules excluding the outer hyperechoic rim (epineurium/fat). These measurements were obtained three times at both CSAd and CSAp levels and a median value for each site was recorded for statistical analysis (27).

### Statistical analysis

The Quantitative data were expressed as mean and standard deviation (Mean ± SD), Inter-reader agreement on ultrasound measurements was investigated using Cohen’s kappa coefficient between the two ultrasonography readings. For the analytic purpose there was no significant difference between CSAd, CSAp, ∆CSA and Mean CSApd, (*p > 0.05*) measurements in the right and left wrists measurements of patients with bilateral CTS, so each wrist assumed as independent variable for analysis. The CTS group and healthy controls group were compared using a *t* test for age, CSAd, CSAp, mean CSAdp and the ΔCSA measurements. χ^2^ test for subject sex. The comparison in relation to neurophysiologic severity the CSAd, mean CSAdp and the ΔCSA were compared using a *t* test. Pearson correlation coefficients were computed for measurements of CSAd, mean CSAdp and ΔCSA to determine whether there was any significant correlation between measurements obtained from the right and left wrist measurements in patients with bilateral CTS evaluation, also correlation coefficients were done to assess the relation of CSAd, mean CSAdp and ΔCSA measurements with the median nerve neurophysiological grades of severity.

The accuracy and cutoff values of US in the diagnosis of CTS were tabulated by using a receiver operating characteristic (ROC) analysis for CSAd, mean CSAdp and ΔCSA measurements.

Analysis of variance (ANOVA) was used to test the differences in ΔCSA measurements in relation to different CTS etiologies (idiopathic, diabetes and hypothyroidism). All statistical tests were performed with (SPSS) version 20 (Armonk, NY: IBM Corp.), and *P* < 0.05 indicated a significant difference.

### Ethics

The study conforms to the 1995 Helsinki declaration and was approved by the ethical committee of Al Hada Armed Forces Hospital, KSA. Written consent form was taken from all patients prior to their inclusion.

## Results

42/60(70%) patients were females and 38/50 (76%) controls were females. The mean patient age was 50.2 ± 11.2 years. 89 wrists of the 60 CTS patients and 76 wrists of the 50 healthy controls were scanned by US,

There was no statistical significant difference regarding age, sex and BMI between CTS patients and controls. However there was a highly significant difference between both groups regarding mean ± SD of CSAd, CSAp, ∆ CSA, and mean CSApd (*p* = 0.0001) (Table 1).

**Table 1:** Demographic characters of the studied group

Variable	CTS patients number of scanned wrists (89)	Control group number of scanned wrists(76)	*P*-value
Sex %	42/60 (70%)	38/50 (76%)	0.14
Age Mean and SD	50.2 ± 11.2 years	48.4 ± 10.2 years	0.8
BMI kg/m^2^	25.4 ± 4.8	24.3 ± 4.6	0.2
CSAd Mean and SD	18.4 ± 5.4	9.3 ± 1.6	0.0001
CSAp Mean and SD	9.3 ± 2.7	8.4 ± 1.6	0.01
∆ CSA Mean and SD	9.2 ± 4.6 mm2	0.9 ± 1.5 mm2	0.0001
Mean CSAdp	14.5 ± 4.2 mm2	8.8 ± 1.5 mm2	0.0001

Carpal tunnel syndrome (CTS), cross sectional area distal (CSAd), cross sectional area proximally (CSAp), cross sectional area difference (ΔCSA), mean average of cross sectional area distal and proximal (CSApd), body mass index (BMI), standard deviation (SD), *P* value was calculated by independent t-test between two groups with 95%CI

Severe CTS according to neurophysiological assessment was found in 31 wrists with 58 wrists diagnosed as mild to moderate. Cohen’s kappa test showed highly statistical inter-reader agreement regarding both ultrasonography measurements (k = 0.88, *p* < 0.001). There was a statistical significant difference in CSAd values between mild to moderate CTS (16.4 ± 5.1 mm^2^) and severe CTS (19.7 ± 5.3 mm^2^) (*P* = 0.01), additionally there was a highly statistical significant difference in ∆CSA values between mild to moderate CTS (5.8 ± 3.1 mm^2^) and severe CTS (11 ± 4.3 mm^2^) (*P* = 0.001), furthermore there was a highly statistical significant difference in the mean of CSApd values between mild to moderate CTS (8.8 ± 1.5 mm^2^) and severe CTS (14.4 ± 4.2 mm^2^).

A positive significant correlation was also found between the CSAd, ∆ CSA and mean of CSApd measurements with the severity grade based on the neurophysiologic studies (P = 0.001) (Table 2).

**Table 2:** Correlation of CTS neurophysiological Severity in Nerve Conduction Examination and ultrasound measurement

Correlation with NCS grade of severity	r	p-value
CSAd	0.278	0.008
∆ CSA Mean and SD	0.644	0.0001
Mean CSAdp	0.293	0.005

Cross sectional area distal (CSAd), cross sectional area difference (ΔCSA), mean average of cross sectional area distal and proximal (CSApd), nerve conduction study (NCS), P value was calculated by Pearson correlation test

**Fig. 4:**
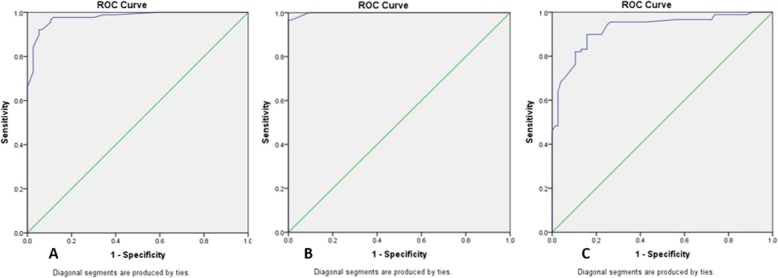
The receiver operating characteristic (ROC) curve for CSAd (A), ∆CSA (B) and Mean CSAdp (C) measurements in CTS patients

To assess the accurate cutoff value and the most sensitive and specific threshold values, a receiver operating characteristic (ROC) curve was done and the area under the curve was calculated. The ultrasound measures of CSAd with area under the curve = 0.978, showed threshold values of 11, 12, and 13 mm^2^, the ∆CSA with area under the curve = 0. 998, showed threshold values of 2, 2.5, 3 mm^2^, and mean CSAdp with area under the curve = 0.925, showed threshold values of 9.5, 10, 10.5 mm^2^ were illustrated in Table 3. The best diagnostic values were achieved by using a ∆CSA threshold of 2.5 mm^2^. Receiver operating characteristic analysis revealed excellent ability to use CSAd, ∆CSA, and Mean CSAdp (Fig. 4, Table 3).

**Table 3:** Sensitivity and Specificity of Nerve Measurements in the Diagnosis of CTS

Measurement	Sensitivity (%)	Specificity (%)
CSAd 11 mm2 threshold	97	89
CSAd 12 mm2 threshold	92	94
CSAd 13 mm2 threshold	90	95
∆CSA with 2 -mm2 threshold	97	90
∆CSA with 2.5-mm2 threshold	97	100
∆CSA with 3-mm2 threshold	96	100
Mean CSAdp with 9.5-mm2 threshold	90	81
Mean CSAdp with 10 -mm2 threshold	83	85
Mean CSAdp with 10.5-mm2 threshold	82	90

Cross sectional area distal (CSAd), cross sectional area difference (ΔCSA), mean average of cross sectional area distal and proximal (CSApd),

58 scanned CTS wrists were characterized as idiopathic, 18 diabetes-associated CTS and 13 hypothyroidism-associated CTS. There was no significant difference regarding ∆CSA between diabetic, idiopathic and hypothyroidism CTS groups (*P* > 0.05), however there was statistical significant difference regarding mean CSAdp and CSAd values within groups (*P* < 0.05), Post Hoc analysis was done showed no significant difference regarding mean CSAdp and CSAd values between idiopathic and hypothyroidism associated CTS or between hypothyroid and diabetes associated CTS. However a significant difference was found between mean CSAdp and CSAd values between diabetes and idiopathic CTS, with higher measurement values in diabetic patients (P < 0.05) (Table 4).

**Table 4:** Comparative study of CTS ultrasonography measurement in relation to different etiology

Ultrasound measurement	Mean and SD	Etiology	P-value
Idiopathic CSAd	17.59 ± 4.84	Diabetes	**0.002**
		Hypothyroidism	0.6
Diabetes CSAd	22.01 ± 6.5	Idiopathic	**0.002**
		Hypothyroidism	0.07
Hypothyroidism CSAd	18.5 ± 4.47	Idiopathic	0.057
		Diabetes	0.07
Idiopathic ∆CSA	8.52 ± 4.55	Diabetes	0.08
		Hypothyroidism	0.5
Diabetes ∆CSA	11.2 ± 5.1	Idiopathic	0.08
		Hypothyroidism	0.3
Hypothyroidism ∆CSA	9.48 ± 3.75	Idiopathic	0.5
		Diabetes	0.3
Idiopathic Mean CSAdp	13.57 ± 3.37	Diabetes	**0.02**
		Hypothyroidism	0.07
Diabetes Mean CSAdp	16.48 ± 5.24	Idiopathic	**0.02**
		Hypothyroidism	0.6
Hypothyroidism Mean CSAdp	15.76 ± 4.67	Idiopathic	0.07
		Diabetes	0.6

Cross sectional area distal (CSAd), cross sectional area difference (ΔCSA), mean average of cross sectional area distal and proximal (CSApd), P value was calculated by ANOVA test

## Discussion

Median nerve entrapment in the wrist joint is the most frequent entrapment neuropathy [1, 28], with high prevalence in females [29]. US is a noninvasive tool that can assess the median nerve in CTS [5, 6]. We investigated the use of US CSA measurements of the median nerve in patients with electrophysiological confirmed CTS and in non-affected normal controls to validate this technique in the Saudi population. We evaluated the difference and average mean of both the proximal, non affected, part of the median nerve at the level of the pronator quadratus muscle and the CSA at the wrist where you would expect to find the maximum enlargement due to distal compression effect, [20]. Furthermore, we calculated the mean of CSA proximal values to omit the individual variability rather than depend on CSA at the level of carpal tunnel only.

We found a statistical significant difference in the US values of CSAd, CSAp, ∆CSA, and mean CSAdp between CTS patients and healthy controls, with these findings was in agreement with Klauser et al. [20].

Although neurophysiologic study is accurate in diagnosis of CTS [1], US has the added value of potentially identifying the underlying pathology causing CTS, such as anatomical variants or space occupying pathology [3–6]. In the our study, the CSAd was statistically correlated to the severity of CTS with neurophysiologic assessment, the higher the value the more severe the CTS, which were concordant with other studies [20, 30, 31]. However, Mhoon et al. did not find a significant correlation between CSAd and electrophysiologic severity assessment [32]. Klauser et al. also [20] stated that ∆CSA is correlated with neurophysiologic CTS severity, which agreed with our findings related to ∆CSA. In addition, we found that the mean CSAdp also statistically correlated to the severity of CTS which was not assessed in previous studies.

Previous studies attempted to ascertain a universal cutoff value of CSA for the diagnosis of CTS, however until now there was no standard universal cutoff range, being mainly dependent on the ethnicity of the group studied [7–12]. In previous literature, the suggested values of CSAd median nerve abnormality varies between 9 to 14 mm^2^ [27, 28]. Although discrepancies in the accuracy of sonographic criteria of median nerve entrapment have been reported, CSA with cutoff values of more than 9 or 10 mm^2^ at the scapho-pisiform level indicating CTS [12–15] is still agreed upon as the most reliable and reproducible sonographic criterion indicating CTS [33], with sensitivity of 82% and specificity of 87% (which were almost equal to those of electrodiagnostic values) with a cutoff CSA of more than 12 mm^2^ considered as excellent to diagnose CTS [11]. In the current work, the cutoff value of CSA at the level of carpal tunnel to diagnose CTS in Saudi population was 13 mm^2^ with 90% sensitivity and 95% specificity which was in agreement with other studies cutoff threshold that ranged between 12 and 13 mm^2^ in CTS with or without diabetes [20, 34, 35]. The mean CSAdp of 9.5 showed sensitivity of 82 and 95% specificity to diagnose CTS, however ∆CSA of 2.5 mm^2^ was associated with 97% sensitivity and 100% specificity, with much higher sensitivity and specificity than using CSAd and mean CSAdp. In another study, the ∆CSA of 2 mm^2^ or greater was considered diagnostic for CTS with sensitivity of 99% and a specificity of 100% [20].

Electrodiagnostic testing is considered the reference standard for CTS diagnosis with sensitivities of 82–94% and specificities of 65–97% [10–12, 15], even though paresthesia may occur before changes can be measured with nerve conduction tests [36]. US is advantageous in that it is noninvasive, less painful (and therefore more acceptable to patients), more readily available and less expensive. According to our results, US is highly sensitive and specific, so that it may be used as an early tool to assess CTS in patients that may have negative or borderline electrophysiologic studies since negative electrophysiologic studies may occur in up to 30% of CTS patients [9]. According to our results, ultrasound is highly sensitive and specific, so it may be used as an early tool to assess CTS where patients may have negative or borderline electrophysiologic studies.

In the current study, we also looked at different CTS etiologies. There was a statistical difference regarding CSAd, and mean CSAdp values between CTS patients with idiopathic CTS and diabetes, however, there was no statistical difference between different etiologic groups regarding ∆CSA. A recent meta-analysis on CSA of CTS with diabetes, in spite being not statistically significant (*p* = 0.52), CSA of median nerve in patients with diabetes and CTS were likely to be larger than patients with idiopathic CTS [17]. A study by Thomsen et al. found focal CSA enlargement of median nerve in diabetic patients without CTS, particularly at the level of the carpal tunnel inlet, they suggested other additional factors which may contribute to such phenomenon, such as a reduction in myelinated nerve fibers and capillary density that may predispose diabetics to develop CTS [37]. Imaging modalities as US and magnetic resonance imaging (MRI) have had a significant role in understanding, assessment and diagnosis of disorders of the peripheral nervous disorders [39].

Our study had several limitations. The assessment of US measurements CSAd, ∆CSA and mean CSAdp in cases with bifid median was not done on our group of patients. In addition, this study is only assessed in one ethnic group with smaller sample size.

Strengths of the current study are that it was designed as a case controlled study involving a less commonly studied ethnic group, the inclusion of disease severity and various etiologies, the correlation of the different US measurements to disease severity, the sub analysis of US measurements in relation to variable etiologies and the establishment of a cutoff diagnostic value of CTS in Saudi population with ultrasound.

## Conclusion

Ultrasound assessment in carpal tunnel syndrome is noninvasive diagnostic tool with correlation to the degree of entrapment severity with a ∆CSA greater than 2.5 mm^2^ being considered diagnostic for CTS in Saudi population. Diabetic CTS patients tend to have higher median nerve US measurements values as opposed to idiopathic and hypothyroid CTS in our study group. This study, in our opinion, emphasizes the importance of utilizing US in the diagnosis of early stage CTS to alleviate permanent nerve damage and functional disability, however, large randomized control studies are needed to assess the current study measurements in bifid median nerve with CTS.

## Data Availability

The datasets used and/or analysed during the current study are available from the corresponding author on reasonable request.

## Abbreviations

BMI: Body mass index
CMAP: Compound muscle action potential
CSAd: Cross sectional area distal
CSAp: Cross sectional area proximally
CSApd: Mean average of cross sectional area distal and proximal
CTS: Carpal tunnel syndrome
DML: Distal motor latency
MRI: Magnetic resonance imaging
NCS: Nerve conduction study
ROC: Receiver operating characteristic
SD: Standard deviation
SNAP: Sensory nerve action potential
SNCV: Sensory nerve conduction velocity
US: Ultrasound
ΔCSA: Cross sectional area difference
